# Psychological State Among the General Chinese Population Before and During the COVID-19 Epidemic: A Network Analysis

**DOI:** 10.3389/fpsyt.2021.591656

**Published:** 2021-02-26

**Authors:** Fenfen Ge, Anni Zheng, Mengtong Wan, Guan Luo, Jun Zhang

**Affiliations:** ^1^Mental Health Center, West China Hospital, Sichuan University, Chengdu, China; ^2^National Laboratory of Pattern Recognition, Institute of Automation, Chinese Academy of Sciences, Beijing, China; ^3^Wuyuzhang Honors College, Sichuan University, Chengdu, China

**Keywords:** COVID-19, psychological state, network analysis, longitudinal study, general population

## Abstract

**Background:** The infectious disease Coronavirus Disease 2019 (COVID-19) outbroke in 2019 spread to multiple countries. The quick spread of the virus and isolation strategies may trigger psychological problems. Our aim was to explore the dynamic network structure of the psychological state before and during the epidemic.

**Methods:** A web-based survey was conducted in two stages: the T1 stage (1 January 2019 to 31 December 2019) and the T2 stage (1 February 2020 to 8 March 2020). In both stages, the Patient Health Questionnaire-9, General Anxiety Disorder-7, and Pittsburgh Sleep Quality Index were used to assess depression, anxiety, and sleep, respectively.

**Results:** We matched the data based on IP addresses. We included 1,978, 1,547, and 2,061 individuals who completed the depression, anxiety, and sleep assessments, respectively, at both stages. During epidemics, psychomotor agitation/retardation, inability to relax, restless behavior, and the frequency of using medicine had high centrality. Meanwhile, the network structure of psychological symptoms becomes stronger than before the epidemic.

**Conclusion:** Symptoms of psychomotor agitation/retardation, inability to relax, and restless behavior should be treated preferentially. It is necessary to provide mental health services, including timely and effective early psychological intervention. In addition, we should also pay attention to the way patients use medicines to promote sleep quality.

## Introduction

In December 2019, an outbreak of the Coronavirus Disease 2019 (COVID-19) suddenly outbreak (1). The World Health Organization (WHO) defined COVID-19 as a pandemic on 30 January 2020 (2). As the COVID-19 pandemic influences numerous facets of our society, it also impacts each person in different ways. The general population is already feeling disruptions to daily life. Students must stay at home and are not allowed to attend school. Factories and companies have closed, and the comforting social gatherings that usually fill weekends are off limits. We are feeling uncertain about what could happen in the coming weeks, as we hope to slow the spread of the COVID-19.

Infectious diseases [e.g., severe acute respiratory syndrome (SARS) and COVID-19] and the related containment measures have negative influence on individuals physically and mentally. During the SARS epidemic, higher stress levels, poor sleep, and depressed mood were reported among confirmed cases (3). Fiorillo and Gorwood's (4) and Brooks et al.'s (5) studies found that the quarantine, social distancing, and self-isolation would reduce the social interactions and increase in loneliness (4, 5). Huang et al.'s (6) study found that the incidence of anxiety in medical staff was 23.04% and that there were higher levels of posttraumatic stress symptoms during the COVID-19 epidemic. Ge et al.'s (7) study found that the prevalence rate of probable anxiety and probable insomnia was 12.49 and 16.87% among undergraduate students, respectively. In addition to special groups (confirmed cases, medical staff, and students), COVID-19 has triggered a variety of psychological problems (e.g., anxiety and depression) among the general public (8). Moccia et al.'s (9) research indicated 19.4 and 18.6% of the Italian general population in their survey shown mild and moderate-to-severe likelihood of psychological distress.

Mental health has an irreplaceable role in managing infectious diseases like COVID-2019. Unreasonable emotional reactions may exacerbate disease spread in pandemic areas (10). Thus, it is necessary to pay attention to mental health (e.g., depression, anxiety, and sleep problems) among the general population and provide suitable psychosocial support and prevention strategies.

Borsboom and Cramer (11) and Borsboom (12) have proposed a network theory. Based on this novel perspective, symptoms are consistent with mental disorders. Network structure consists of two elements: nodes and edges. Every node represents a symptom, and each edge demonstrates a relationship between two symptoms. Nodes have different importances in the network. High centrality nodes have stronger connections to many other nodes and have greater effects on the network structure. In addition, high centrality symptoms play a key role in the development, persistence, remission, and relapse of mental disorders (13). Centrality is commonly evaluated with four indices: expected influence, strength, closeness, and betweenness. Nodes do not appear in isolation, and symptoms tend to co-occur if they have a positive edge (14). An event external (COVID-19) to the general population may activate one or more nodes (e.g., fear, insomnia) that in turn activate another based on the strength of the edge linking them (15).

We identified three limits in previous studies on psychological state after sudden public health events. First, most of the studies are cross-sectional studies and only focus on mental health status during or after public health events. A significant drawback of these studies is the ignorance of the intrinsic levels of depression, anxiety, or sleep symptoms. Second, no studies have explored the network structure of psychological state structure during or after public health events. Previous studies only found that the incidence rate of anxiety and/or depression increased among the general population but did not evaluate the relationship between these symptoms. Network analysis provides a new perspective on understanding the connection between symptoms. Third, relatively few studies have focused on the psychological responses of infectious diseases (SARS and COVID-19) among the general Chinese population (8, 16, 17). Liu et al.'s (18) study found that 23 surveys concern medical staff, 18 surveys concern students, and 9 surveys concern the general population.

We present the first study to use a network model to explore the dynamics of depression, anxiety, and sleep symptoms before and during the COVID-19. Our goal is to explore whether centrality symptoms and global connectivity have changed and provide a general population with more targeted psychological intervention and support.

## Materials and Methods

### Study Sample

Participants were recruited from the WeChat of China online social media platform. The mental health survey was placed on WeChat and targeted all Chinese users aged 18 years or older. The survey was implemented online using a Haola applet, with data stored on a secure server at Sichuan University. The study attained approval from the West China Hospital, Sichuan University. The target population of WeChat users aged ≥18 years was 1 billion, representing ~76.9% of the total Chinese population aged ≥18 years. Data collection was divided into two stages: the T1 stage (1 January to 31 December 2019) and the T2 stage (1 February to 8 March 2020). The IP addresses contain accurate location information. With the IP address matched, T1 and T2 groups have the same location distribution.

### Measures

#### Patient Health Questionnaire-9

The Patient Health Questionnaire-9 (PHQ-9) is a self-report questionnaire that efficiently evaluates depression based on DSM-IV criteria. It includes only 9 items and takes <3 min to complete (19). A study has shown that the PHQ-9 has good reliability and validity in Chinese (20). In this study, the Cronbach's alpha value was 0.644 at T1 and 0.674 at T2.

#### General Anxiety Disorder

The General Anxiety Disorder (GAD-7) is a valid and efficient instrument screening for anxiety symptoms and assessing its severity in both clinical practice and research. Higher scores indicate more severe anxiety symptoms. Evidence also supports its good reliability, criterion, construct, factorial, and procedural validity among Chinese people (21). In this study, the Cronbach's alpha value was 0.686 at T1 and 0.713 at T2.

#### Pittsburgh Sleep Quality Index

The Pittsburgh Sleep Quality Index (PSQI) is a self-rated questionnaire that evaluates sleep quality and disturbances. It only takes 2–5 min to complete. Studies have found the PSQI to be reliable and valid in the assessment of self-reported sleep problems among Chinese people (22). In this study, the Cronbach's alpha value was 0.691 at T1 and 0.771 at T2.

### Data Analysis

#### Missing Data

When using the Haola applet to fill out questionnaires, you can submit the questionnaires only after completing the information completely. Thus, there are no data missing in the research.

#### Network Estimate

We used R-software (qgraph packages) to estimate the network. The symptoms of depression, anxiety, and sleeping problems were described as nodes, and the correlations between symptoms were described as edges in the network. According to the guidelines of Epskamp and Fried (23), the gLASSO program (conservatively identifying the relevant edges only and discovering the underlying network structure accurately) was used to calculate the network. The estimated value of every edge can be understood as a partial correlation, which represents a unique relationship between two symptoms that were independent of all other symptoms (11). Not all the edges are showing in the network graph. We use the threshold presented by Cao et al. (24) to omit edges, which lead to a low false-positive rate and sparser network graph. In addition, we used Extended Bayesian Information criterion (EBIC) for parameter tuning and then chose the best network estimation. In the present analysis, all variables are detected as ordinal, so polychoric correlations can be calculated automatically by using the cor_auto function. Visualization of the network is accomplished by the Fruchterman-Reingold algorithm.

#### Centrality Estimation

We calculated four node centralities that included closeness, betweenness, strength, and expected influence. We considered that node betweenness and node closeness are often not reliably estimated (25). Node strength cannot reflect the negative relationships between symptoms (13). Thus, we only reported the expected influence in this article, and other node centralities are provided in the Supplementary Material.

#### Network Stability

We evaluated the stability of the network from two aspects: edge weight stability and node centrality stability. We estimated the edge weight stability by bootstrapping the 95% confidence intervals (CI). The fewer overlaps in the CIs show higher stability. We measured the node centrality stability by the correlation stability coefficient (CS coefficient). According to Epskamp's study, to better gain a stable and interpretable centrality, the CS coefficient should be >0.25 and 0.5 (26).

#### Network Comparison

Global connectivity was conducted using the Network Comparison Test (NCT). NCT is a common estimation method for all kinds of data and networks (27). Global strength, which is defined as the weighted absolute sum of all edges of the network, summarizes the overall connectivity.

## Results

### Descriptive Statistics

We included 1,978, 1,547, and 2,061 individuals who completed depression, anxiety, and sleep assessments, respectively, at two stages. The basic characteristics are shown in Table 1. Table 2 demonstrates the mean symptom severity scores for each symptom at T1 and T2.

**Table 1:** The basic characteristics.

	**Depression**	**Anxiety**	**Sleep**
Gender (male/female)	1,456/522	1,091/456	1,389/672
Age	32.50 ± 10.64	29.56 ± 9.58	27.21 ± 7.16

**Table 2:** Mean symptom severity scores at T1 and T2.

**Symptoms**	**Mean (SD) scores**	
	**T1**	**T2**
**Depression symptoms**		
Anhedonia (C0)	1.41 ± 0.93	1.56 ± 0.82
Depressed mood (C1)	1.67 ± 0.77	1.68 ± 0.70
Sleep disturbance (C2)	1.70 ± 0.65	1.78 ± 0.57
Low energy (C3)	1.56 ± 0.89	1.56 ± 0.84
Change in appetite (C4)	1.34 ± 0.95	1.36 ± 0.88
Worthless (C5)	1.24 ± 0.97	1.33 ± 0.90
Low concentration (C6)	1.38 ± 0.92	1.46 ± 0.80
Psychomotor agitation/retardation (C7)	1.25 ± 1.02	1.37 ± 0.93
Suicide ideation (C8)	1.38 ± 0.97	1.48 ± 0.87
**Anxiety symptoms**		
Nervous (B0)	1.44 ± 0.81	1.50 ± 0.82
Uncontrollable worry (B1)	1.34 ± 0.91	1.42 ± 0.88
Worry about many things (B2)	1.26 ± 0.93	1.36 ± 0.91
Unable to relax (B3)	1.50 ± 0.79	1.62 ± 0.74
Restless behavior (B4)	1.18 ± 0.90	1.30 ± 0.90
Irritability (B5)	1.20 ± 0.96	1.38 ± 0.93
Fear of awful events (B6)	1.32 ± 0.86	1.44 ± 0.84
**Sleep symptoms**		
Difficulty falling asleep (A0)	2.32 ± 1.00	2.36 ± 1.01
Easy wake up (A1)	2.68 ± 0.76	2.70 ± 0.76
Go to the toilet frequently (A2)	2.00 ± 0.86	1.93 ± 0.90
Cannot breathe comfortably (A3)	2.09 ± 1.11	2.02 ± 1.15
Cough or snore (A4)	0.11 ± 0.53	0.13 ± 0.56
Feel cold (A5)	2.50 ± 0.86	2.49 ± 0.90
Feel hot (A6)	1.34 ± 1.09	1.32 ± 1.07
Nightmare (A7)	1.65 ± 0.91	1.44 ± 1.07
Somatic discomfort (A8)	1.37 ± 1.17	1.33 ± 1.13
Other reasons influence you sleep (A9)	2.39 ± 0.95	2.40 ± 0.99
The frequency of using medicine (A10)	2.68 ± 0.75	2.72 ± 0.72
Feel sleepy (A11)	2.14 ± 1.06	2.10 ± 1.08

### Network Estimation

**Figure 1:**
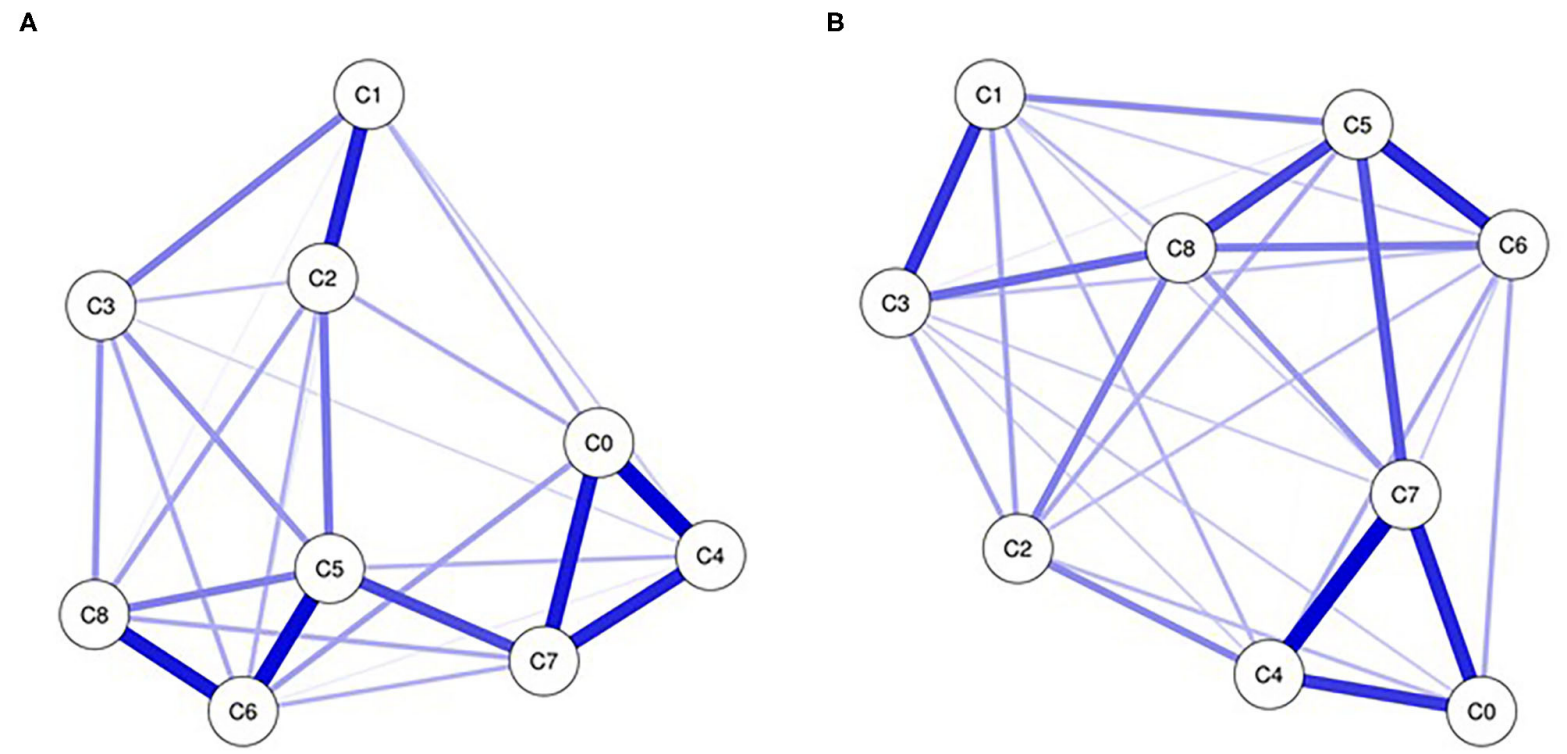
Networks of depression at T1 **(A)** and T2 **(B)**. Nodes present depression symptoms and edges present partial connections between symptoms. Edge darkness and thickness present the connection strength. Edge color demonstrates the association valence (blue, positive; red, negative). C0, anhedonia; C1, depressed mood; C2, sleep disturbance; C3, low energy; C4, change in appetite; C5, worthless; C6, low concentration; C7, psychomotor agitation/retardation; C8,suicide ideation.

The networks of depression symptoms at the T1 and T2 stages are shown in Figures 1A,B, respectively. The two networks featured many consistent edges, such as the strong relationships between anhedonia, change in appetite and psychomotor agitation/retardation (C0:C4:C7), and the moderate relationship between worthless and psychomotor agitation/retardation (C5:C7). Some specific edges that differed across the two networks, such as depressed mood and low energy (C1:C3) and worthless and suicidal ideation (C5:C8), were very weak at T1 but strong at T2.

**Figure 2:**
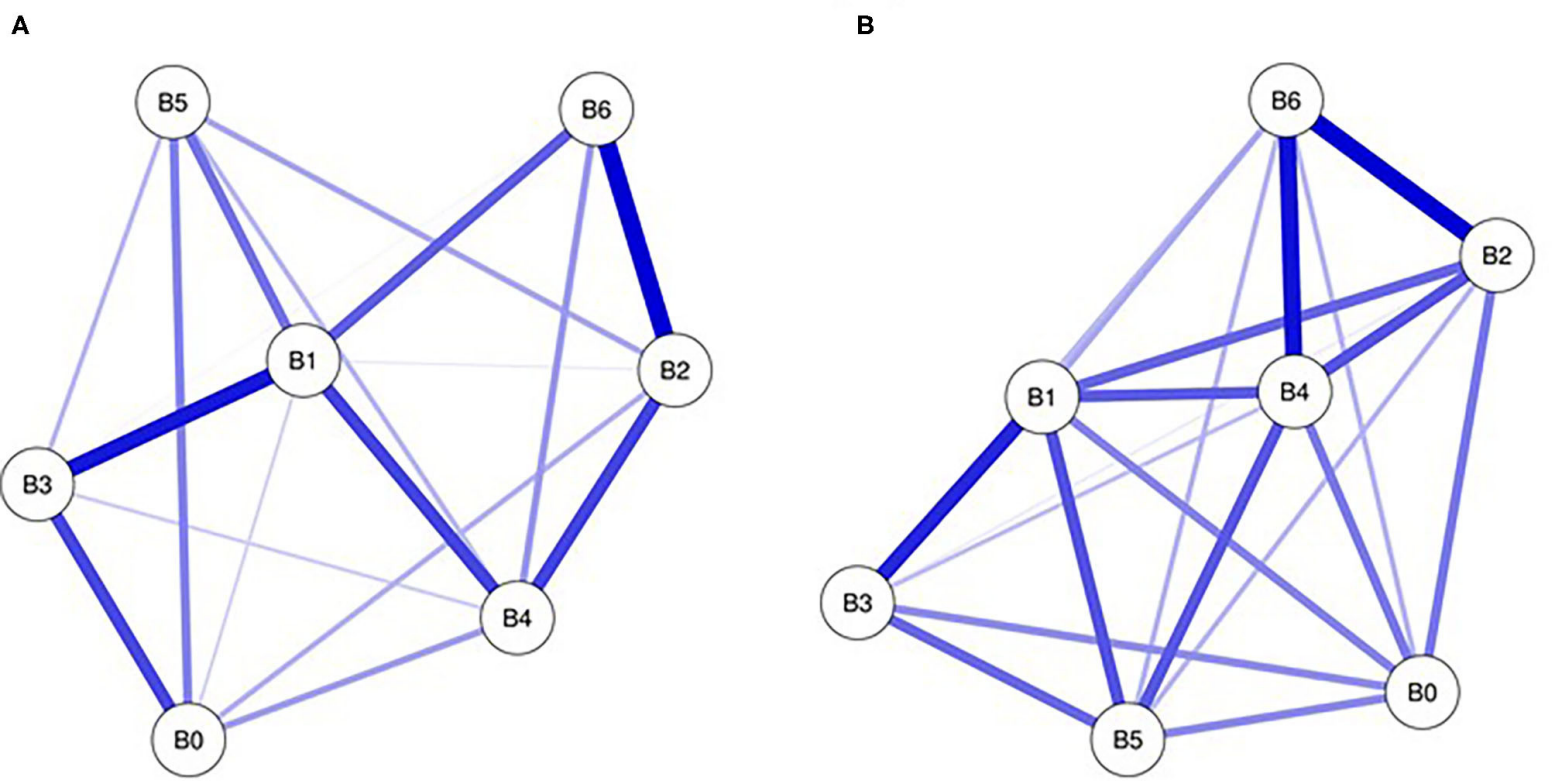
Networks of anxiety at T1 **(A)** and T2 **(B)**. Nodes present anxiety symptoms and edges present partial connections between symptoms. Edge darkness and thickness present the connection strength. Edge color demonstrates the association valence (blue, positive; red, negative). B0, nervous; B1, uncontrollable worry; B2, worry about many thins; B3, unable to relax; B4, restless behavior; B5, irritability; B6,fear of events.

The networks of anxiety symptoms at the T1 and T2 stages are shown in Figures 2A,B, respectively. In two networks, there are strong connections between worry about many things and fear of events (B2:B6) and uncontrollable worry and inability to relax (B1:B3). The connections between restless behavior and fear of events (B4:B6) and restless behavior and irritability (B4:B5) were very weak at T1 but strong at T2.

**Figure 3:**
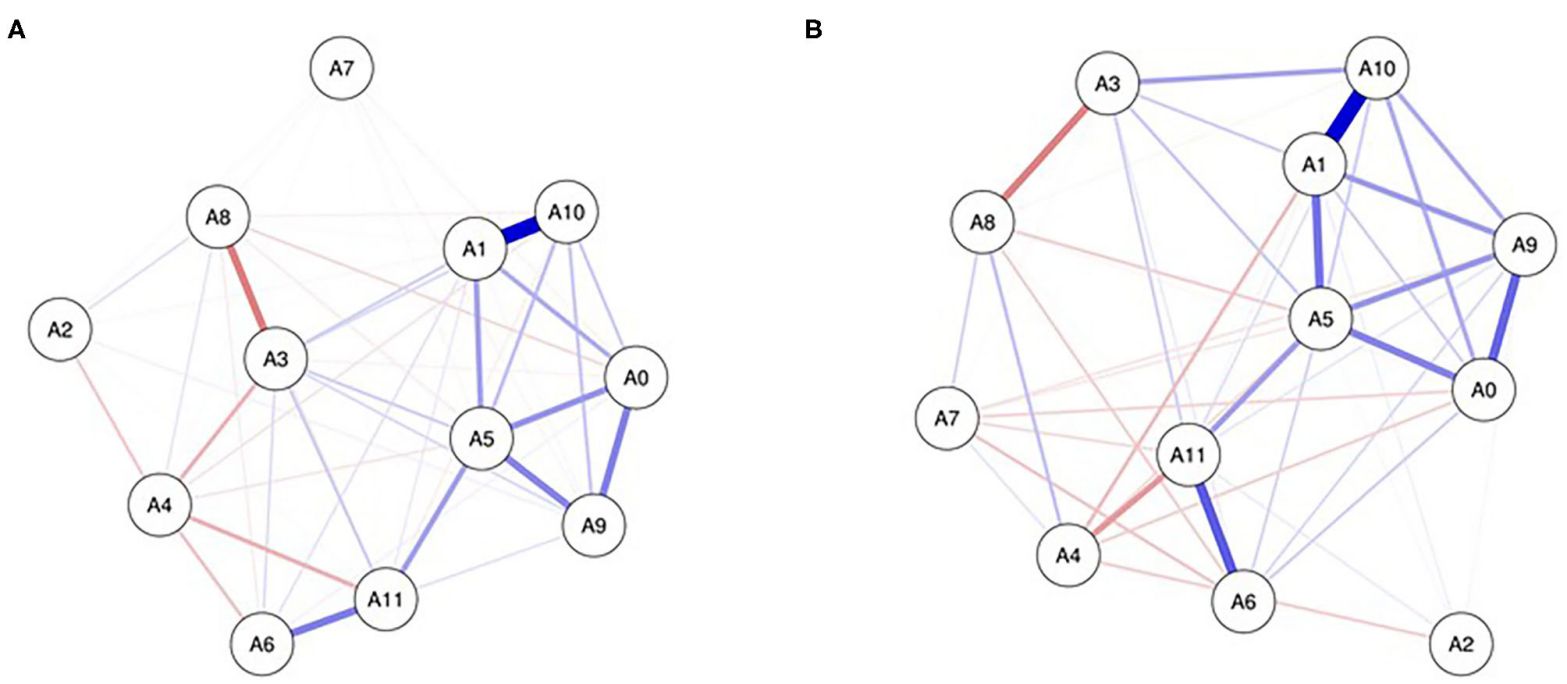
Networks of sleep symptoms at T1 **(A)** and T2 **(B)**. Nodes present sleep symptoms and edges present partial connections between symptoms. Edge darkness and thickness present the connection strength. Edge color demonstrates the association valence (blue, positive, red, negative). A0, difficulty falling asleep; A1, easy wake up; A2, go to the toilet frequently; A3, cannot breathe comfortably; A4, cough or snore; A5, feel cold; A6, feel hot; A7, nightmare; A8, somatic discomfort; A9, other reasons influence you sleep; A10, the frequency of using medicine; A11, felling sleepy.

The networks of sleep symptoms at the T1 and T2 stages are shown in Figures 3A,B, respectively. At the two stages, there is a strong positive connection between easy wake-up and the frequency of using medicine (A1:A10) and a negative connection between the inability to breathe comfortably and somatic discomfort (A3:A8). The connections between feeling hot and feeling sleepy (A5:A11) are weak at T1 and strong at T2. At the T2 stage, the symptoms of easy wake-up, the frequency of using medicine, feeling cold, and difficulty falling asleep (A0:A5:A1:A10) form a closed loop.

### Network Influence and Stability

**Figure 4:**
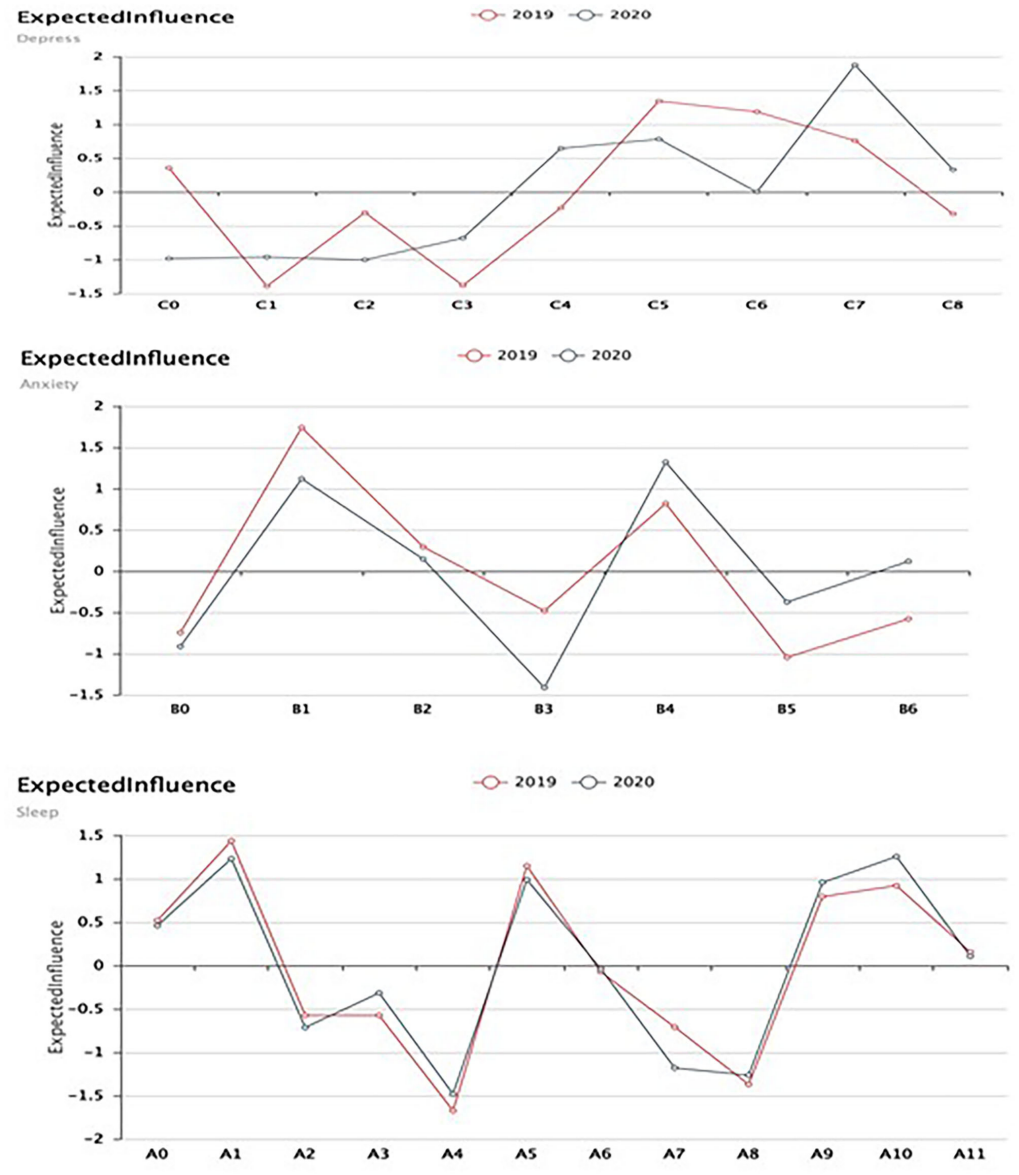
Expected influence of depression, anxiety, and sleep symptoms. C0, anhedonia; C1, depressed mood; C2, sleep disturbance; C3, low energy: C4, change in appetite; C5, worthless; C6, low concentration; C7, psychomotor agitation/retardation; C8, suicide ideation. B0, nervous; B1, uncontrollable worry; B2, worry about many things; B3, unable to relax; B4, restless behavior; B5, irritability; B6, fear of events A0, difficulty falling asleep; A1, easy wake up; A2, go to the toilet frequently; A3, cannot breathe comfortably; A4, cough or snore; A5, feel cold; A6, feel hot; A7, nightmare; A8, somatic discomfort; A9, other reasons influence you sleep; A10, the frequency of using medicine; A11, felling sleepy.

The expected influences of depression, anxiety, and sleep symptoms are shown in Figure 4. At the T1 stage (2019), worthless (C5), uncontrollable worry (B1), easy wake-up (A1), and feeling cold (A5) had high centrality estimates. During the epidemic (2020), psychomotor agitation/retardation (C7), inability to relax (B3), restless behavior (B4), and the frequency of using medicine (A10) had high centrality. The CS coefficients for node centrality were 0.75, 0.21, and 0.44 for depression, anxiety, and sleep, respectively, at T1 and were 0.75, 0.60, and 0.67 for depression, anxiety, and sleep, respectively, at T2. The edge weights of CIs have better stability (Supplementary Material).

### Global Connectivity

The NCT test indicated higher global connectivity in the anxiety network at T2 than at T1 (2.513 vs. 2.543; *p* = 0.029). There was higher global connectivity in the depression network at T2 than at T1 (4.043 vs. 3.814; *p* = 0.229) and higher global connectivity in the sleep network at T2 than at T1 (2.829 vs. 3.027; *p* = 0.198). However, in the two stages of T2 and T1, there is no significant difference between depressed global connectivity and anxious global connectivity.

## Discussion

The psychopathology network offers a new perspective on understanding the dynamics of symptoms. The increase in the incidence rate of depression and anxiety among the general population is an important manifestation of the impact of the COVID-19. The change in dynamic network structure and in core characteristics can also reflect the severity of their impact from another perspective.

Our results demonstrated that high centrality symptoms vary differently before and during the epidemic. Before the epidemic, worthless depression networks (C5) had high centrality, consistent with previous studies (28, 29). During the epidemic, psychomotor agitation/retardation (C7) has a larger influence on the spread of other symptoms and has become a hallmark symptom of depression. Psychomotor agitation/retardation (C7), as bridge symptoms, tightly connects the three dimensions of depression that included cognition (worthless, C5; low concentration, C6; and suicide ideation, C8), somatic symptoms (change in appetite, C4), and mood (anhedonia, C0). Compared with before the epidemic, this loop is connected more strongly. Helplessness, hopelessness, and worthlessness are hallmark symptoms that conceptualized depression. Boschloo's finds that these symptoms are in high centrality with network approach (30). Our result is different from that. The importance of psychomotor agitation/retardation (C7) in the depression network structure may be due to quarantine and lockdown strategies, which were implemented in some countries. Individuals may feel socially isolated, especially if they live alone or are in a community setting that does not allow visitors because of the outbreak. Disruptions to daily life and limited social activities may also lead to boredom (5). Boredom could in turn lead to psychomotor agitation/retardation (C7), that is, boredom and psychomotor agitation/retardation affect each other.

Before the epidemic, uncontrollable worry (B1) had the highest centrality for the anxiety network. This finding is consistent with common conceptualizations of anxiety and uncontrollable worry regarded as core symptoms (31, 32). During the epidemic, symptoms of inability to relax (B3) and restless behavior (B4) play an important role in the network structure. Stronger connections between several anxiety symptoms were observed. In particular, inability to relax (B3) was linked to a variety of other anxiety symptoms. Inability to relax (B3) and restless behavior (B4) may be caused by confusing, stressful times for all of us. We are all feeling uncertain about what could happen in the coming weeks, as we hope to slow the spread of this pandemic. In this survey, the average age of the participants was ~30 years old. In the Chinese culture, people at this age not only have the responsibility of raising their children but also have the responsibility for taking care of their parents. The sudden epidemic has forced them to stay at home, and reduced income may leave them in a state where they are unable to relax (B3).

There is no study to explore the network structure of sleep symptoms. Before and during the epidemic, we found that easy wake-up (A1) and the frequency of using medicine (A10) have a positive connection. Symptoms of easy wake-up (A1) and feeling cold (A5) also have high centrality. During the epidemic, symptoms of easy wake-up, the frequency of using medicine, feeling cold, and difficulty falling asleep (A0:A5:A1:A10) were connected more strongly. It is worth noting that an increasing number of individuals choose to use medicine to promote sleep quality during the epidemic. One possible explanation is that psychological changes are often accompanied by increased sleep disturbance. During this unique period, to alleviate the psychical and/or psychological problems caused by sleep disturbance, individuals are more inclined to use medicines.

During the epidemic, overall global connectivity was greater than before the epidemic, demonstrating larger associations among symptoms. The results suggest that individuals may become more sensitive to the external environment. Before the epidemic, for the general population, most of the symptoms were dormant. Trigger events (COVID-2019) in the external field lead to network activation. Thus, successful early intervention targeting high centrality symptoms would likely prevent the full syndrome of depression/anxiety/sleep disturbance from emerging (33). However, the interpretation of the results should also be cautious. We do not consider Berkson's bias when comparing network structure between samples with different mean symptom severities (34). Berkson's bias may lead to weaker global connectivity among samples affected by more-severe symptoms compared with less-affected samples.

What intervention strategy do the results offer? Network analysis can be used to demonstrate which symptoms should be prioritized. We identified several high centrality symptoms during the epidemic. These results play an important role in developing appropriate treatments for the general population. Compared with the SARS epidemic, during the COVID-2019 outbreak, multiple online mental health services through WeChat/Weibo were provided for the general population (18). To more efficiently allocate limited medical resources and improve the effectiveness of interventions, we can prioritize high centrality symptoms (e.g., psychomotor agitation/retardation, inability to relax, and restless behavior). However, even though suicide ideation has low centrality, it should also be given more attention (35). Barbisch et al.'s (36) study found that the rate of suicidal behavior increased during the SARS epidemic. In addition to intervening nodes, weakening edges and changing circumstances reduce the stressors that may contribute to recovery (37). Specifically, we can also intervene in the connections between symptoms (e.g., the connection between easy wake-up, the frequency of using medicine, and difficulty falling asleep) (37). Although we cannot eliminate external events (COVID-19) immediately, we can increase the transparency of information and allow the general population to have a correct understanding of COVID-19.

Although we cannot eliminate external events (COVID-19) immediately, there are substantial measures that we can take. For the authorities, they should improve transparency of information and allow the general population have a correct understanding of COVID-19. It can obviously help to remove restlessness originated by uncertainty. For psychological professionals, we suggest they could help to provide a more centrally coordinated and more efficient support according to our results. For the general public, according to a recent research (38), we recommend several measures such as chatting with friends online, meditation-based programs, singing, and yoga to release the stress and relax the mind.

There are several limitations in the research. First, we employed the snowball strategy due to limited resources. The snowball strategy was not according to a random selection sample, and participants did not reflect the actual mental health of the general population. Moreover, it is cautious that marital and social status data were only collected on T2 stage. As high level of social support is significantly associated with low levels of depression and anxiety (39) and favorable sleep outcomes (40), marital and social status may influence the symptoms that we evaluated. Thus, more research efforts are needed upon this issue in the near future. Second, we used self-reported questionnaires (PHQ-9, GAD-7, and PSQI) in this research. Response bias and recall bias may exist considering that the general population may have underreported or overreported their depression, anxiety, and/or sleep symptoms. We took some measures to decrease this by keeping uniformity of the data collection method. Although Cronbach's alpha was lower (ranging from 0.6 to 0.7) in our research, it indicates an acceptable level of reliability (41, 42). This may be due to the population's being heterogeneous (42). Third, people with preexisting mental health conditions, people at higher risk for severe illness, and people with underlying health conditions can also confound the results. It is worth mentioning that sense of safety after recovery from COVID-19 may be relevant to the symptoms assessed in our research. However, by 8 March 2020, the number of living cases of COVID-19 in China is 77,759 (43), i.e., <0.06‰ of the national population. Taking our sample size into consideration, we were not likely to include statistically significant participants that had recovered from COVID-19 although the snowball sampling was chosen. The future study may enlarge the sample size and explore the relationship between history of COVID-19 in participants and symptoms. Fourth, our data were taken from different seasons (winter and spring). Some evidence shows that mood disorder may be influenced by seasonality (44, 45), and we did not consider the influence of season on mood state.

## Conclusion

The present research was the first to explore the dynamic network structure of depression, anxiety, and sleep symptoms before and during the COVID-19 epidemic. From the network perspective, we can understand how this epidemic triggered and activated symptoms. The results demonstrated that symptoms of psychomotor agitation/retardation (C7), inability to relax (B3), and restless behavior (B4) should be treated preferentially. In addition, we should also pay attention to the way patients use medicines to promote sleep quality. The first is that sleep disturbance becomes less self-regulating. On the other hand, we also need to think about any psychosocial interventions that can improve sleep and avoid unreasonable use of medicines. Global connectivity is stronger than before the epidemic, which suggests that timely and effective intervention is necessary.

## Data Availability Statement

The raw data supporting the conclusions of this article will be made available by the authors, without undue reservation.

## Ethics Statement

The studies involving human participants were reviewed and approved by the Medical Ethics Committee of Sichuan University. Written informed consent for participation was not required for this study in accordance with the national legislation and the institutional requirements.

## Author Contributions

FG: interpretation of data, drafting the manuscript. AZ: analysis and interpretation of data. MW: drafting the manuscript. GL: acquisition of data. JZ: conception and design of study, revising the manuscript critically for important intellectual content. All authors contributed to the article and approved the submitted version.

## Conflict of Interest

The authors declare that the research was conducted in the absence of any commercial or financial relationships that could be construed as a potential conflict of interest.
