# Supplementary figures and images for: Psychological State Among the General Chinese Population Before and During the COVID-19 Epidemic: A Network Analysis
Source: Front Psychiatry. 2021 Feb 26;12:591656. doi: 10.3389/fpsyt.2021.591656 (PMC7952988; doi:10.3389/fpsyt.2021.591656)

● Bootstrap mean    ● Sample

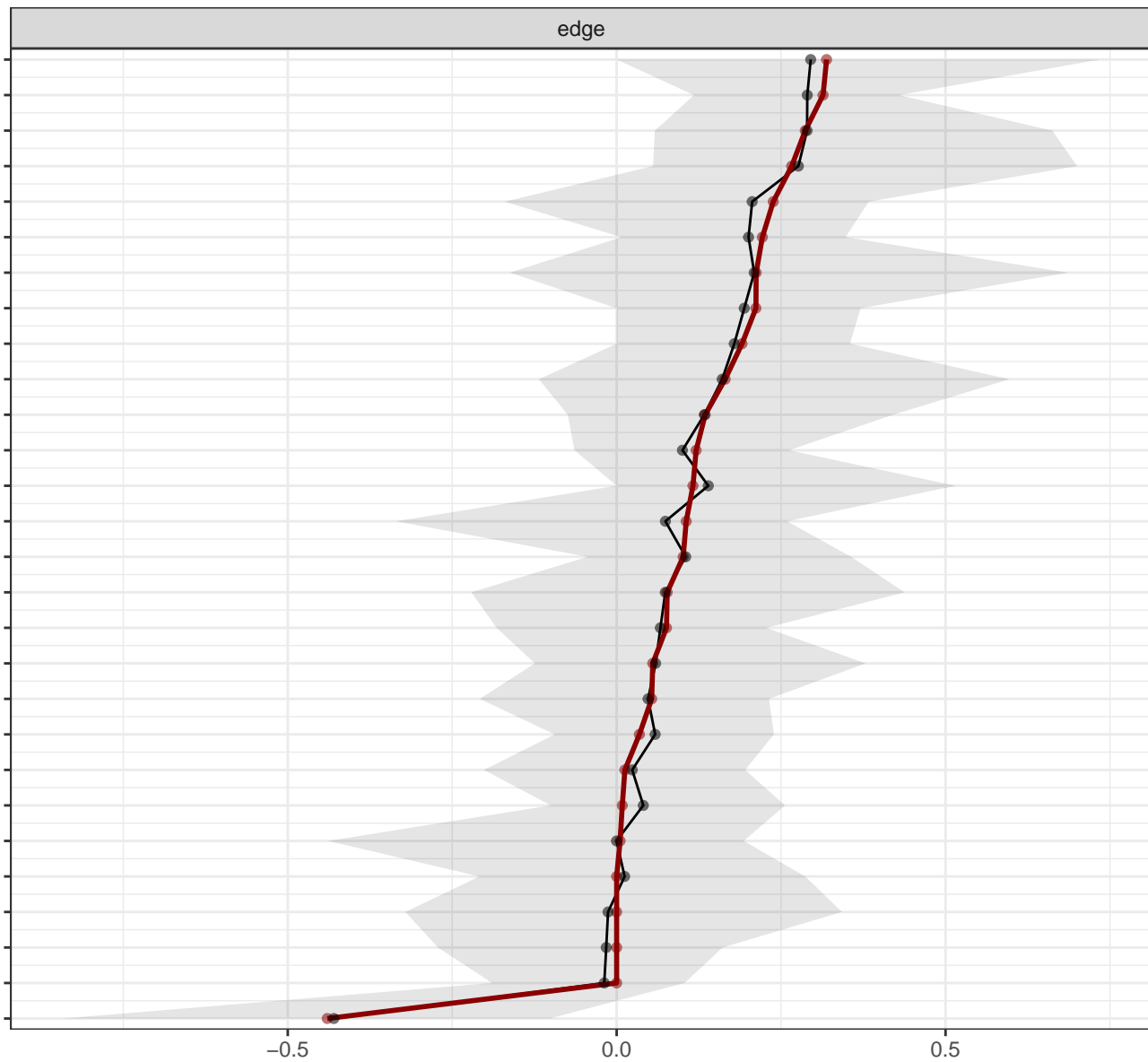

Supplement: Supplementary file 1 [file Data_Sheet_1.PDF]

● Bootstrap mean    ● Sample

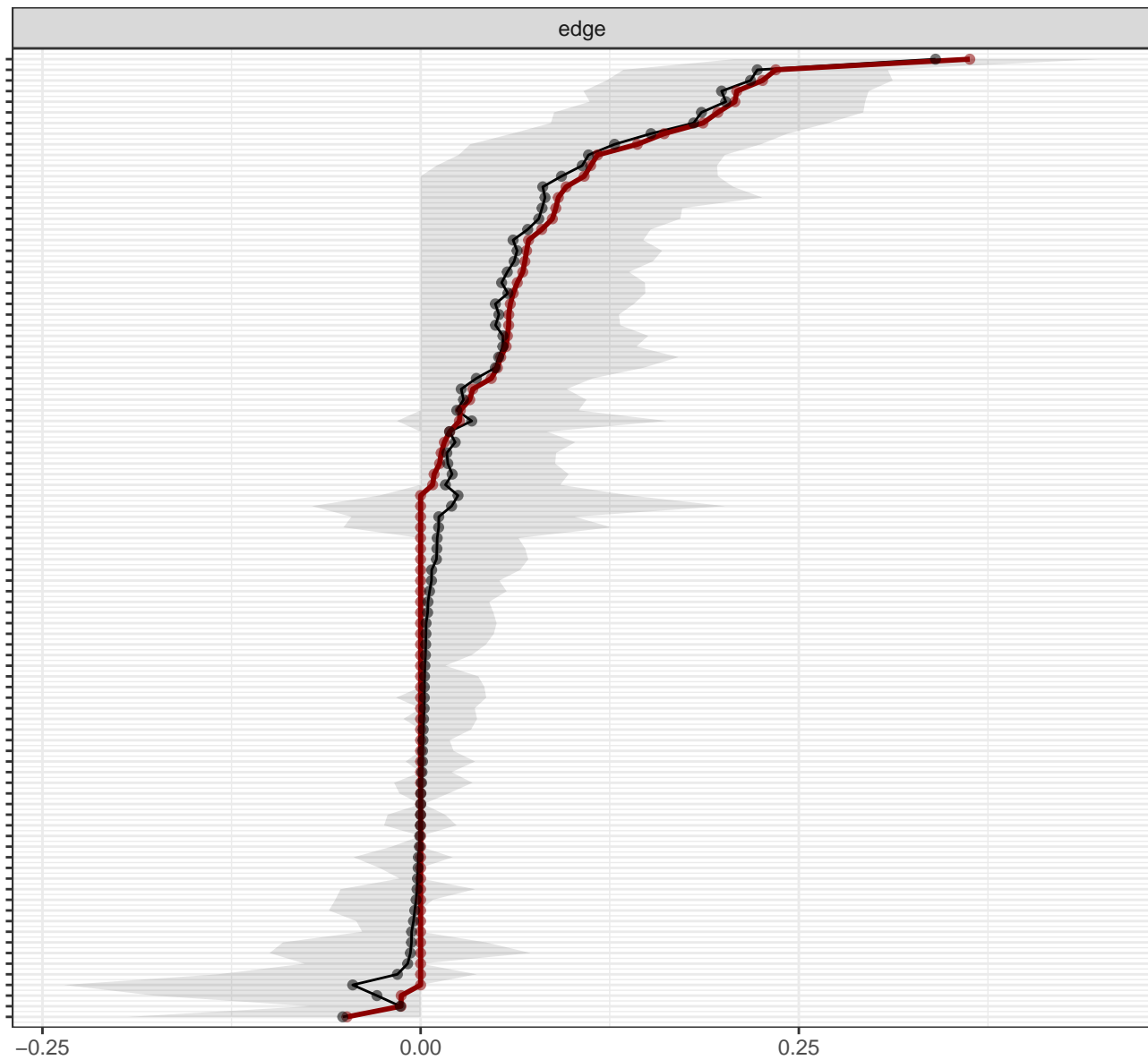

Supplement: Supplementary file 2 [file Data_Sheet_2.PDF]

● Bootstrap mean    ● Sample

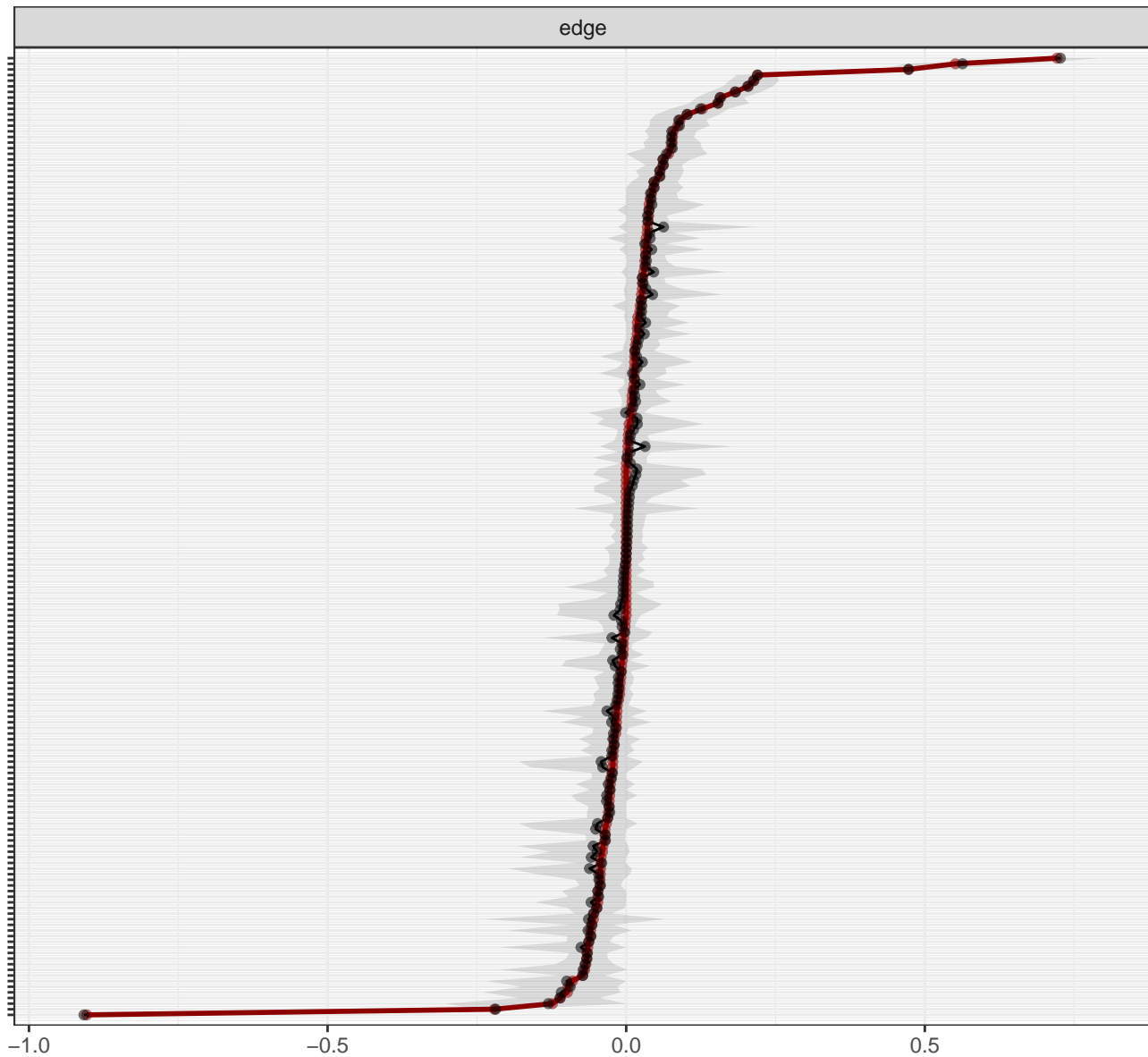

Supplement: Supplementary file 3 [file Data_Sheet_3.PDF]

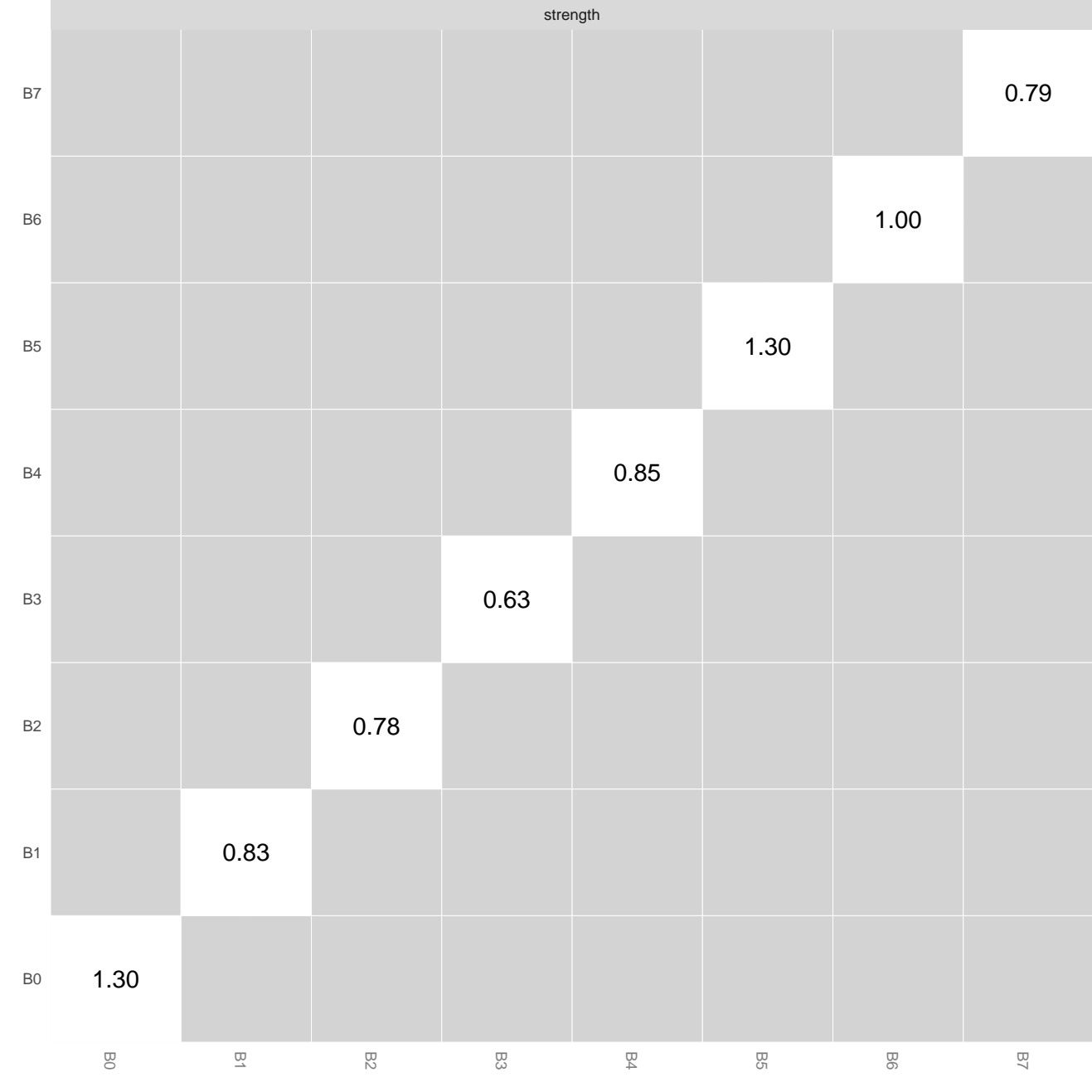

Supplement: Supplementary file 4 [file Data_Sheet_4.PDF]

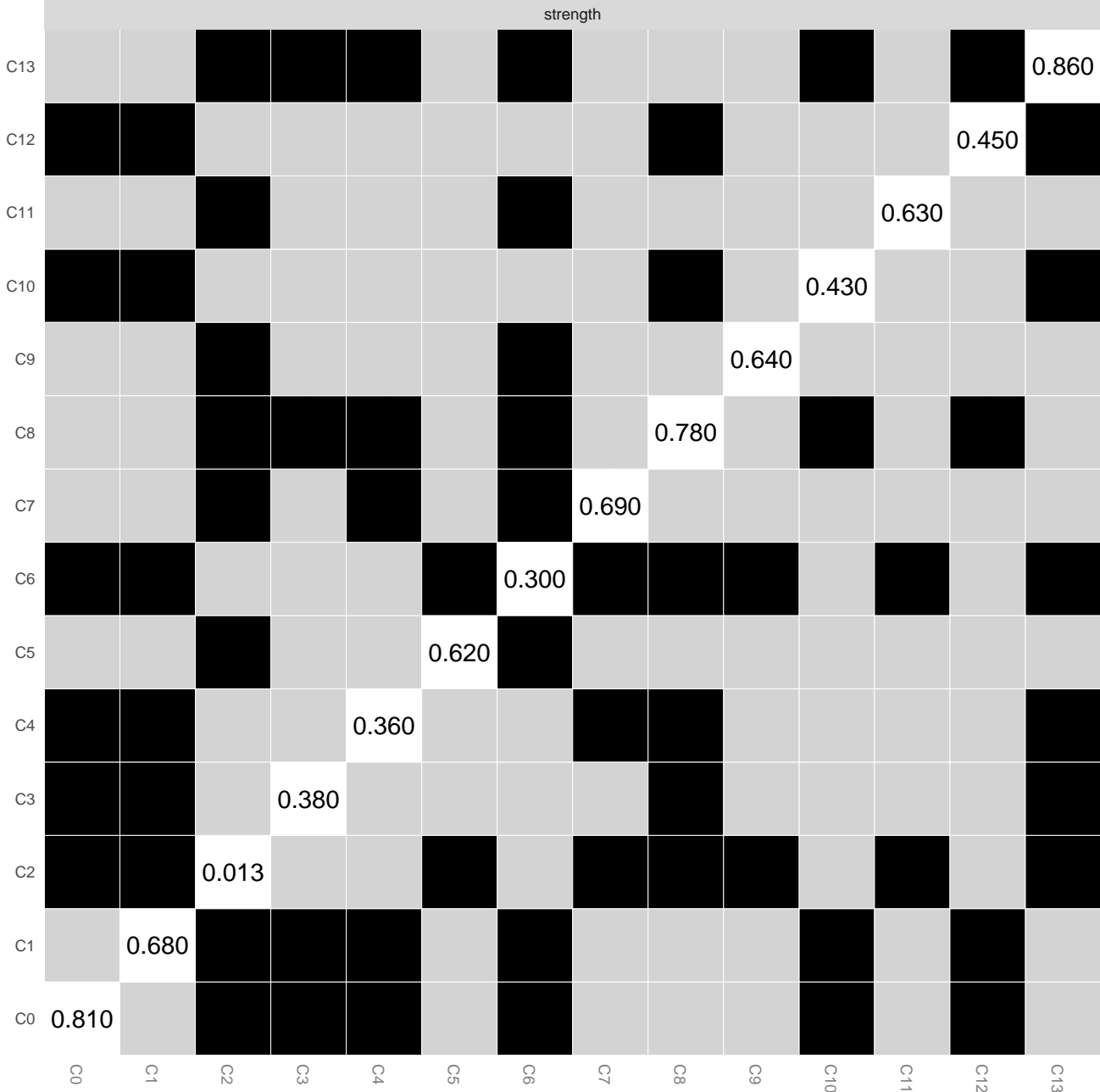

Supplement: Supplementary file 5 [file Data_Sheet_5.PDF]

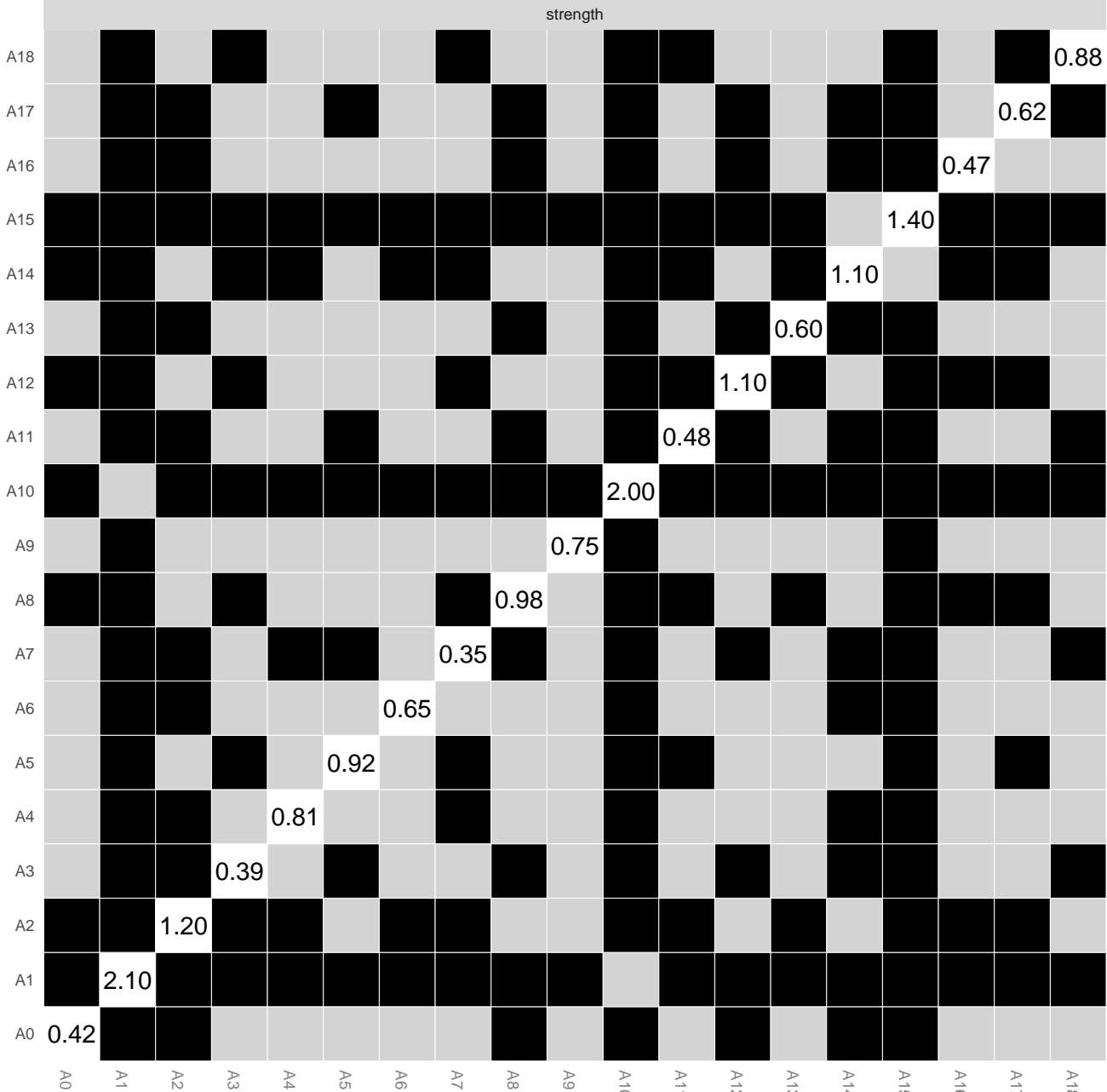

Supplement: Supplementary file 6 [file Data_Sheet_6.PDF]

● Bootstrap mean    ● Sample

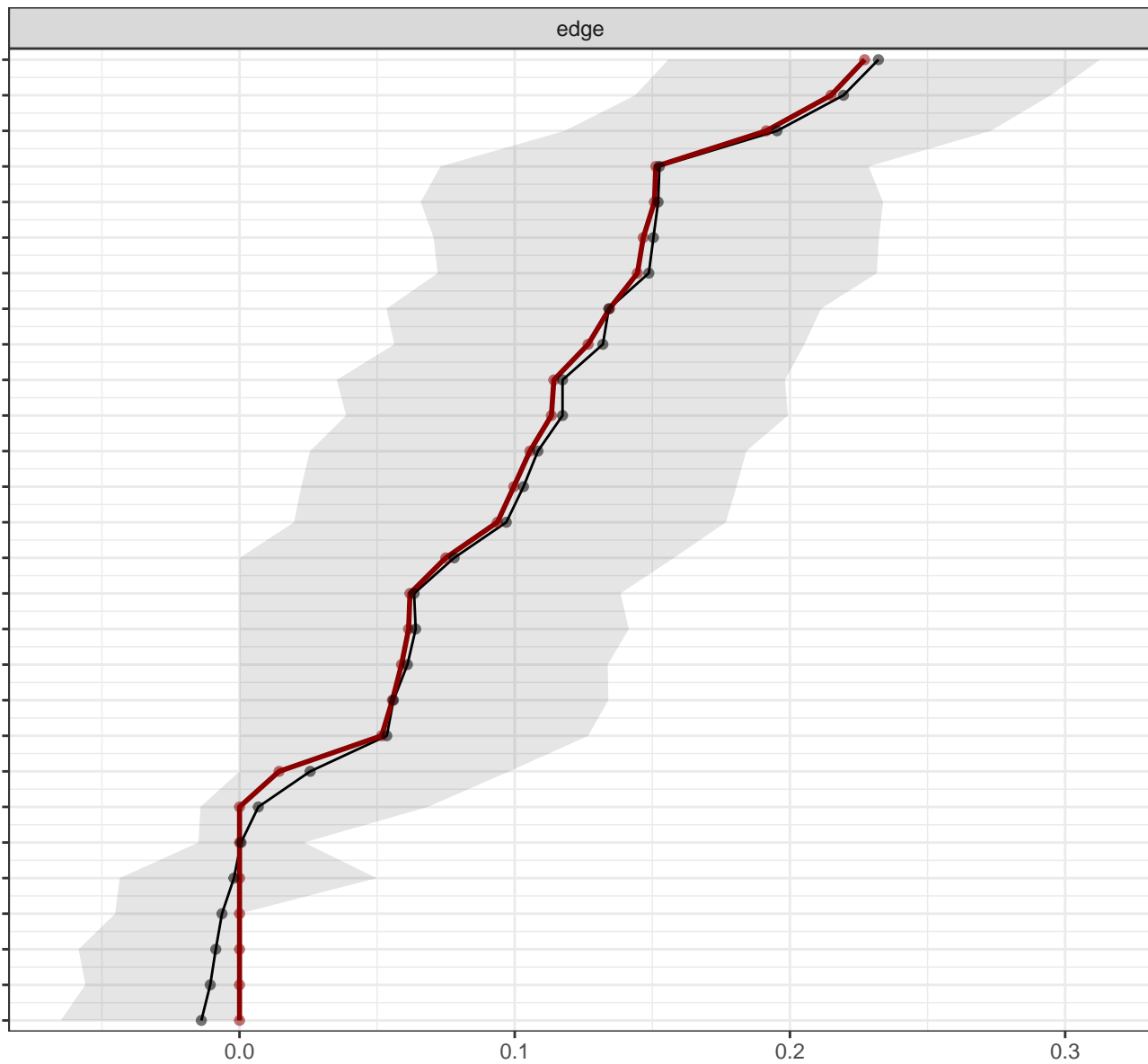

Supplement: Supplementary file 7 [file Data_Sheet_7.PDF]

● Bootstrap mean    ● Sample

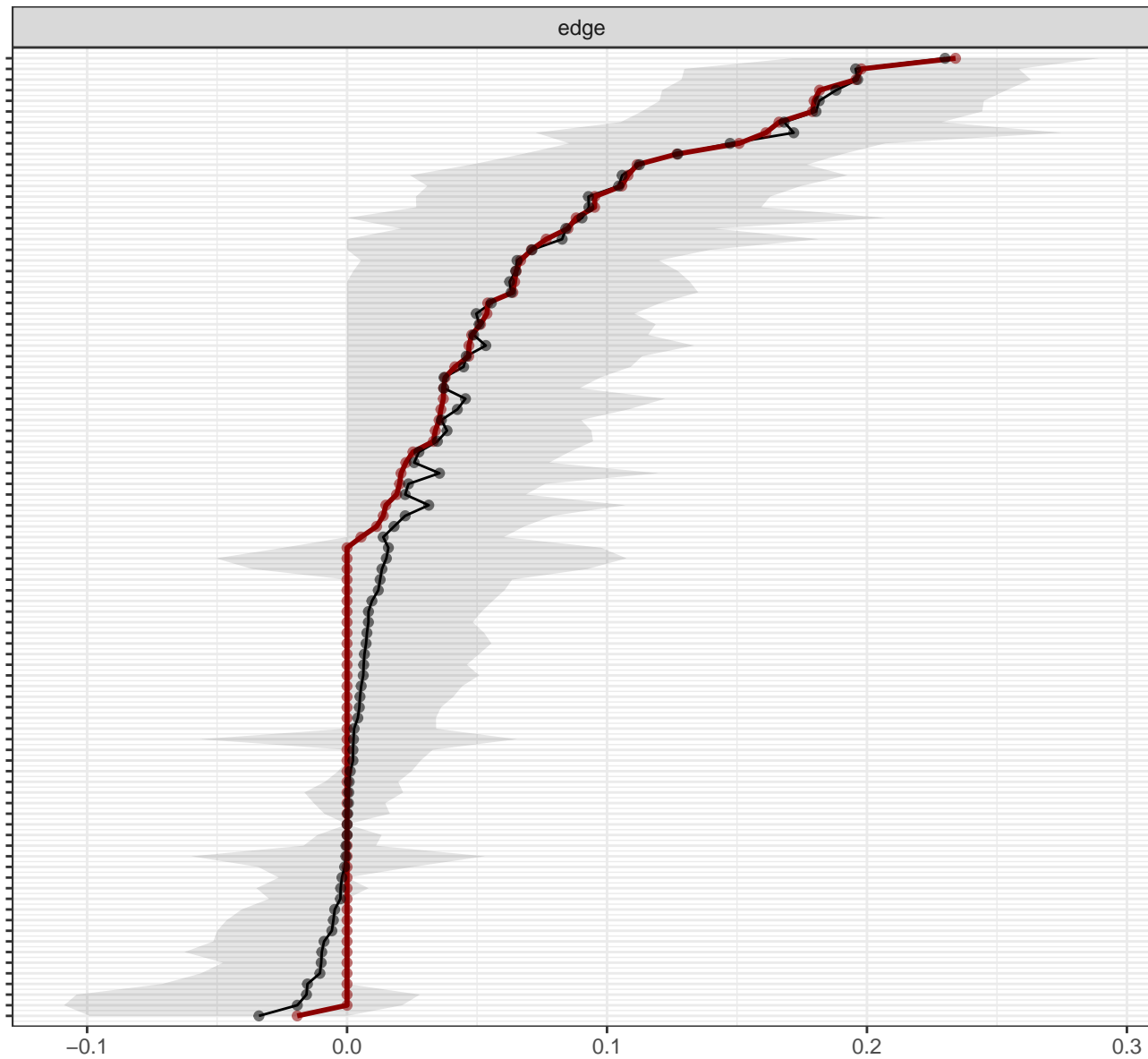

Supplement: Supplementary file 8 [file Data_Sheet_8.PDF]

● Bootstrap mean    ● Sample

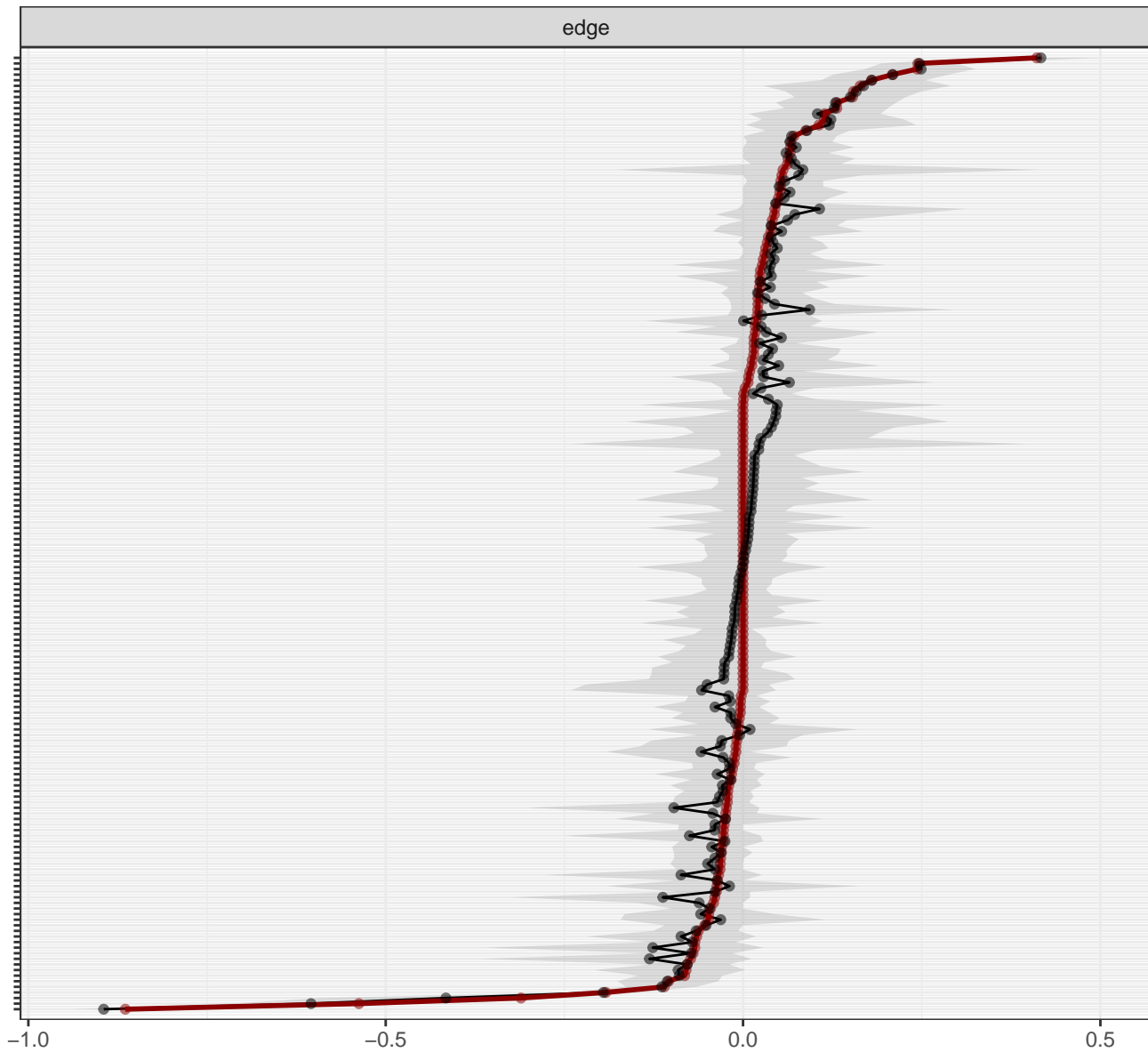

Supplement: Supplementary file 9 [file Data_Sheet_9.PDF]

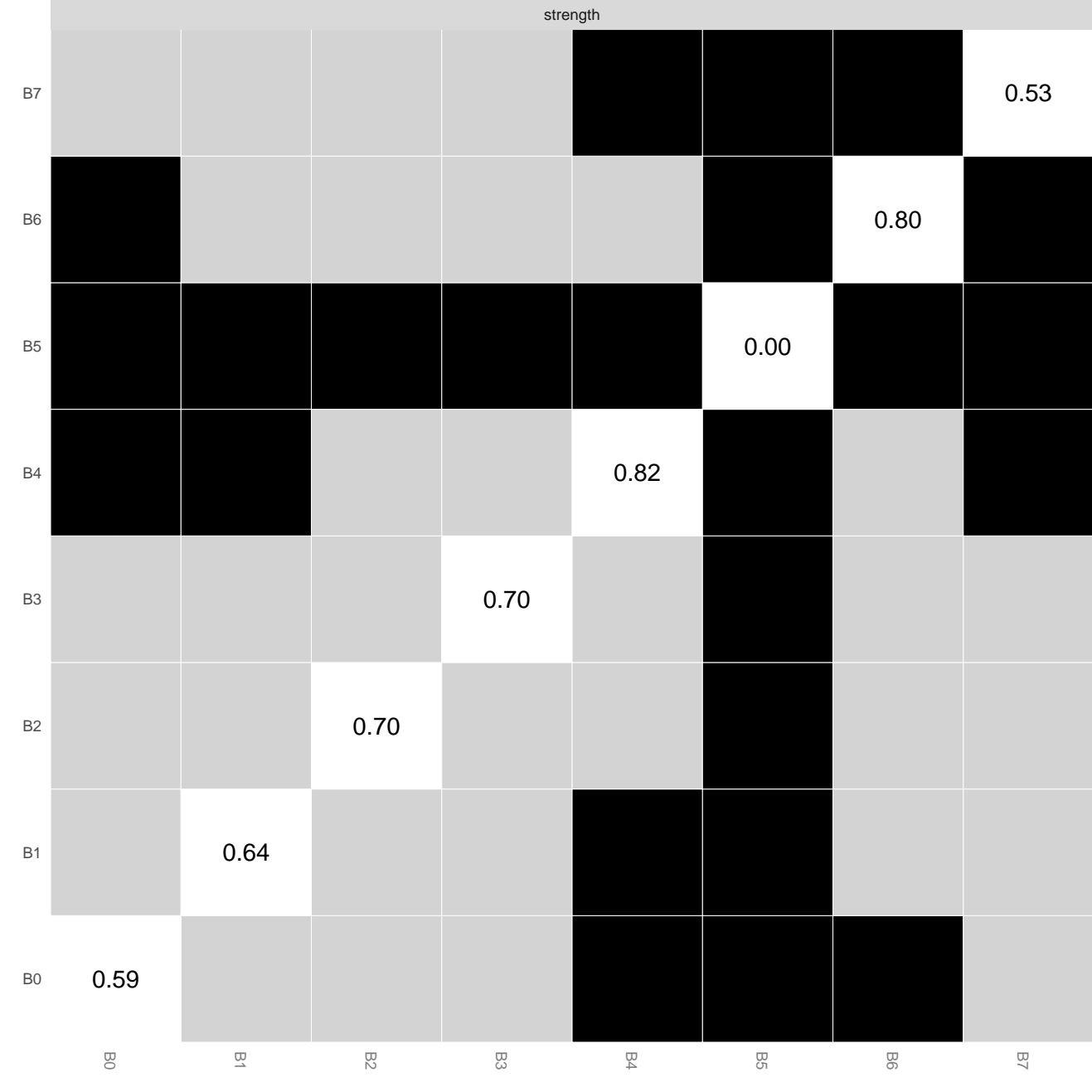

Supplement: Supplementary file 10 [file Data_Sheet_10.PDF]

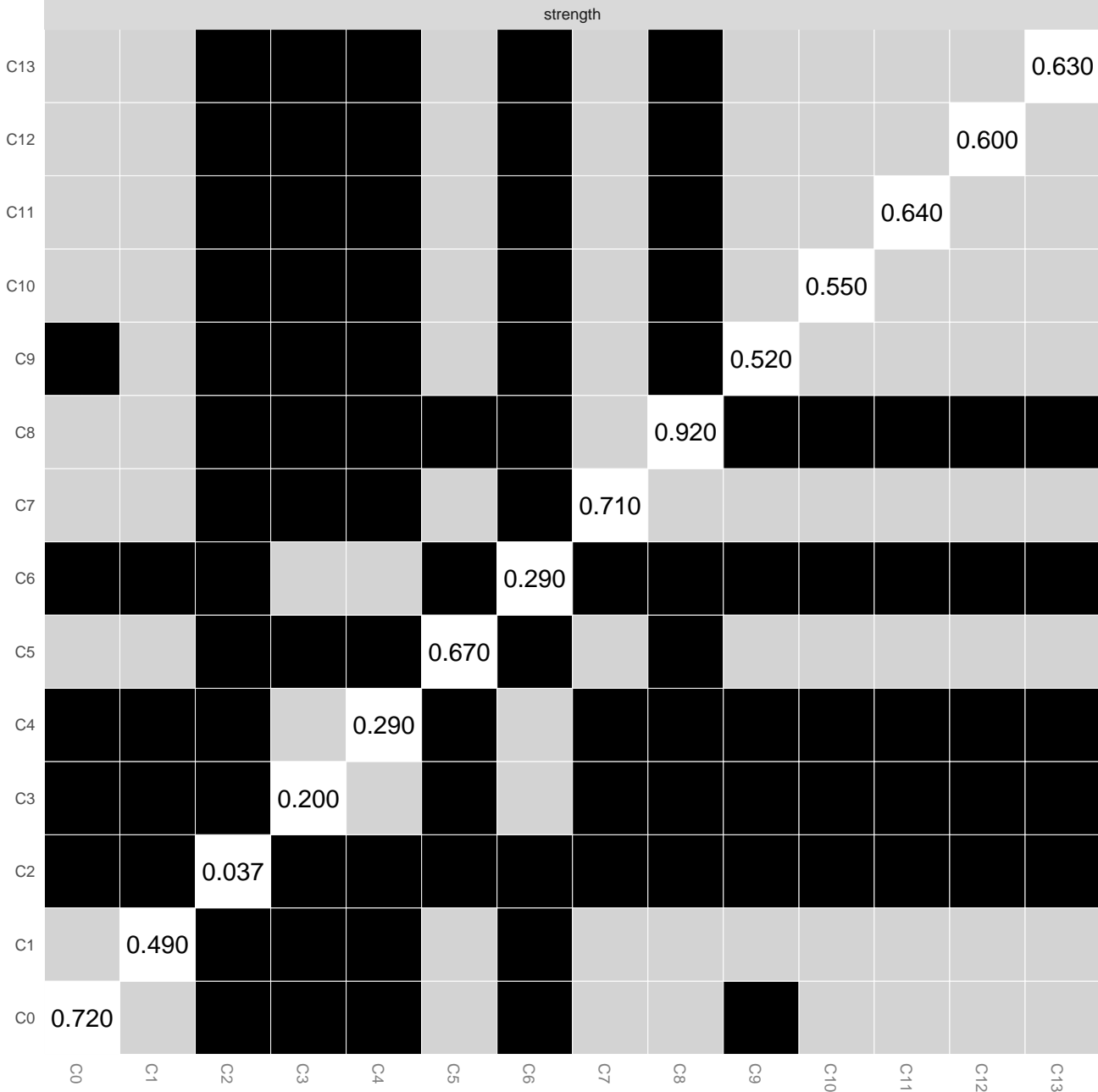

Supplement: Supplementary file 11 [file Data_Sheet_11.PDF]

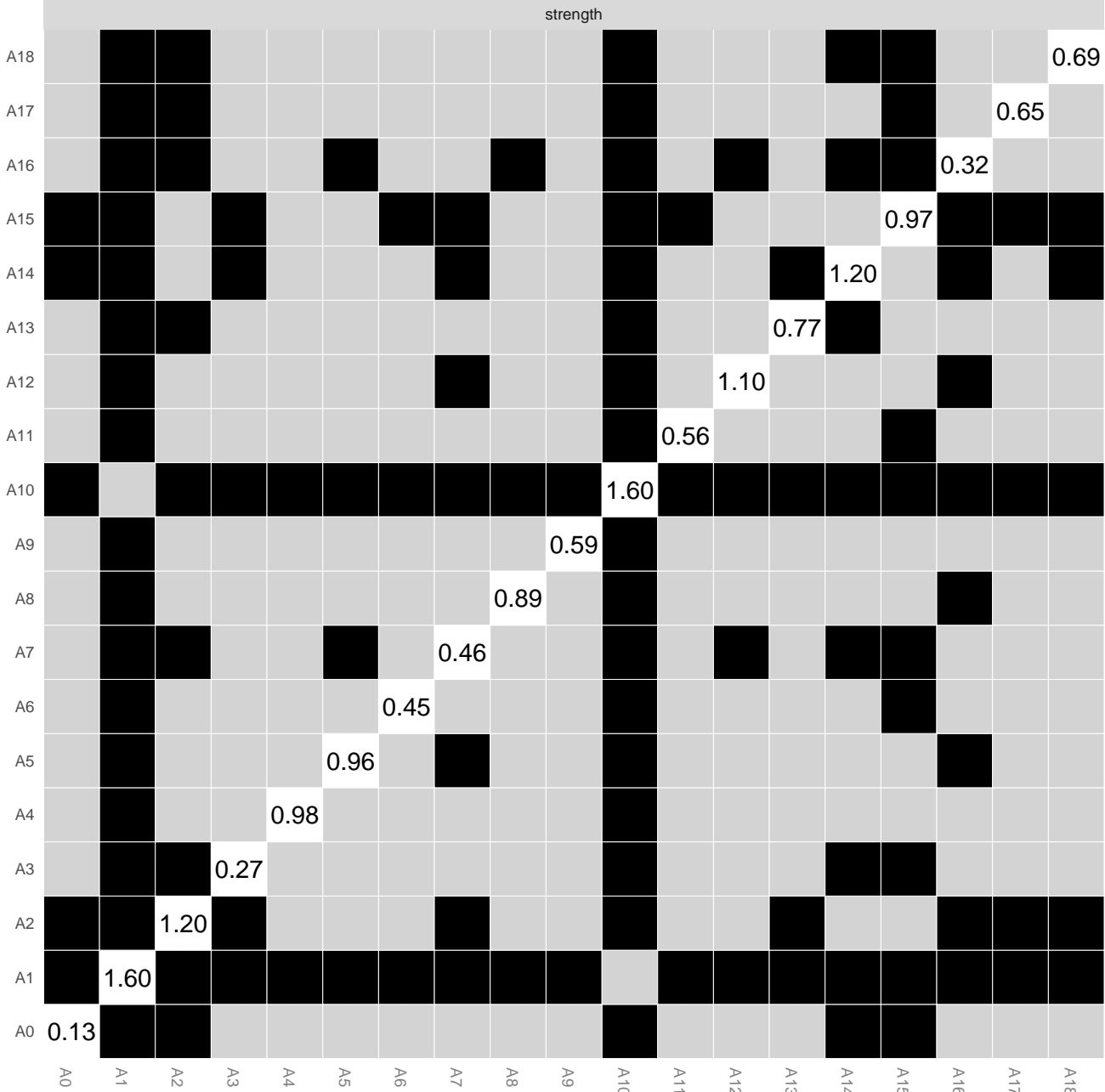

Supplement: Supplementary file 12 [file Data_Sheet_12.PDF]
